# Supplementary material for: Targeting Toxoplasma gondii CPSF3 as a new approach to control toxoplasmosis
Source: EMBO Mol Med. 2017 Feb 1;9(3):385–94. doi: 10.15252/emmm.201607370 (PMC5331205; doi:10.15252/emmm.201607370)
Supplement: Supplementary file 1 — Appendix [file EMMM-9-385-s001.pdf]

Targeting *Toxoplasma gondii* CPSF3 as a new approach

to control toxoplasmosis

Andrés Palencia, Alexandre Bougdour, Marie-Pierre Brenier-Pinchart, Bastien Touquet, Rose-Laurence Bertini, Cristina Sensi, Gabrielle Gay, Julien Vollaire, Véronique Josserand, Eric Easom, Yvonne R. Freund, Hervé Pelloux, Philip J. Rosenthal, Stephen Cusack and Mohamed-Ali Hakimi

Appendix

Appendix Table S1

Appendix Table S1. Oligonucleotide primers used in the gene-editing experiments.

Primer name	Sequence 5' to 3'
TGGT1_285200_F	CAGCGACGGTTTCTCAGGTGAC
TGGT1_285200_R	GGGAACAGAAACCGCTCAAGTG
CPSF3^{E545K}-CRISPR-FWD	AAGTTGTCCTCGTTCACGGAgAGCGG
CPSF3^{E545K}-CRISPR-REV	AAAACCGCTcTCCGTGAACGAGGACA
CPSF3^{E545K}_(s)	GCAGACACAAGAGTTCATTGGGAAGCTGAAGGTCCC<br>GAATGTCGTCCTCGTTCACGGAaAGCGCGGAGAAATGC<br>GCAGACTGAAGGAGAAACTCGAGGAAGAGCGACCCGC<br>GTTGTCTGT
CPSF3^{Y328C}-CRISPR-FWD	AAGTTGAATCCATGTGCACGATGACG
CPSF3^{Y328C}-CRISPR-REV	AAAACGTCATCGTGCACATGGATTCA
CPSF3^{Y328C}_(s)	GCGGAGGTGCCTCCCGTCGACGTCCAACTCCTGATCT<br>GTGAAAGCACTTgCGGAATCCATGTGCACGATGACCG<br>GCAGCTCCGCGAGCGCCGGTTCCTCAAAGCCGTCGTA<br>GATATCGT
CPSF3^{Y483N}-CRISPR-FWD	AAGTTGtACTCGGTGAAGGGGACACG
CPSF3^{Y483N}-CRISPR-REV	AAAACGTGTCCCCTTCACCGAGTaCA
CPSF3^{Y483N}_(s)	GAGATCTTCGAGGCCTGGGCGCCGGATGCGAAGAAC<br>GGTGTGATTCTGACTGGGaACTCGGTGAAGGGGA<br>CACTGGCAGACGAGCTGAAGCGAGAACCCGAGAC<br>GATTCAACTTCCAGAC
SAG1-CRISPR-FWD	AAGTTGGCAGTGAGACGCGCCGTCAG
SAG1-CRISP-REV	AAAACTGACGGCGCGTCTCACTGCCA
HRSAG1-5UTR-CPSF3_F	ACTTCATTATTTCTTCTGGTTTTTTGGCGAGTATGTTTCC<br>GAAGGCAGTGAGACGCGCCGTCTCGTTACAATGC<br>CACTGCTC
HRSAG1-3UTR-CPSF3_R	CCATAGCGCCACATCGCAAGAACGACATCAGTGTGGGC<br>GCGGCAAACACCCCTGCCGTGACTGCTGCTTAACTCC<br>GGCACAC
5UTR-SAG1_F	TTGTGCAGCTTTCCGTTCTTC
SAG1_R	CAGATTTGCCTGTTGGGTGAG
