# Supplementary material for: Targeting Toxoplasma gondii CPSF3 as a new approach to control toxoplasmosis
Source: EMBO Mol Med. 2017 Feb 1;9(3):385–94. doi: 10.15252/emmm.201607370 (PMC5331205; doi:10.15252/emmm.201607370)
Supplement: Supplementary file 2 — Expanded View Figures PDF [file EMMM-9-385-s002.pdf]

Expanded View Figures

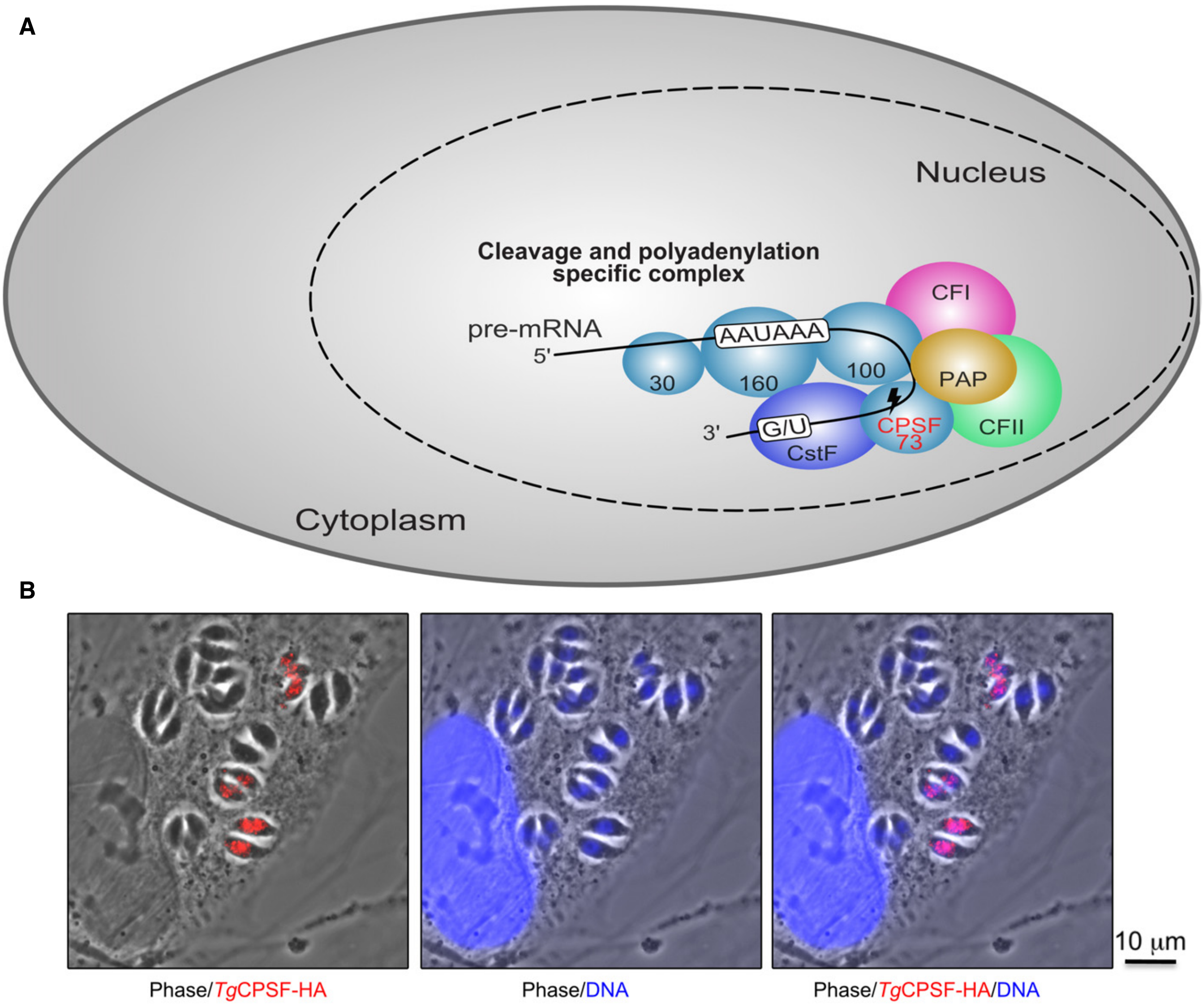

Figure EV1. CPSF3 localizes in the nucleus of *Toxoplasma gondii*.

A Schematic view of the cleavage and polyadenylation specificity complex that, in some eukaryotes, is responsible for processing the newly synthesized pre-mRNAs. CPSF-73, in humans, has the endonuclease activity and acts concertedly with other CPSF subunits, polyadenosine polymerase (PAP) and other factors. Please note that the number of CPSF subunits is dependent on each species and this model is just shown for reference.

B A homologous recombination-proficient *T. gondii* strain (RH *ku80*) was used for endogenous epitope tagging of *T. gondii* CPSF3-HAFLAG (red) hosted in HFF. Staining of CPSF3-HAFLAG was done with anti-HA antibodies and DNA nuclei with Hoechst (blue).

© 2017 The Authors

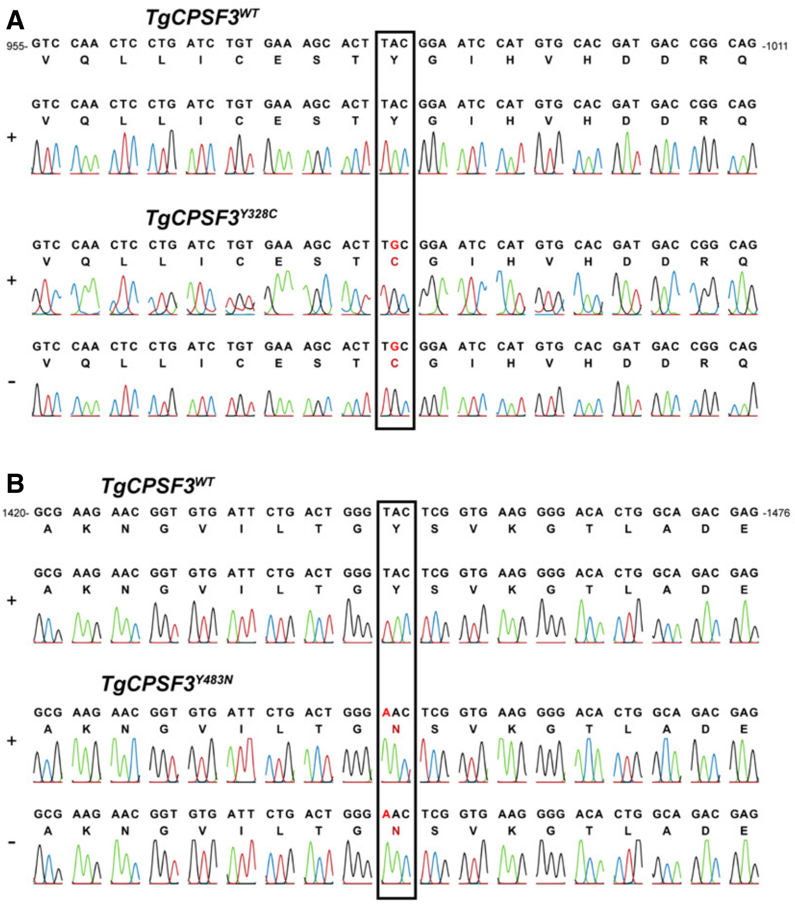

Figure EV2. AN3661-resistant parasites had mutations in *Toxoplasma gondii* CPSF3.

A, B Chromatograms of the sequences of wild-type *Tg*CPSF3 and mutants Y328C (A) or Y483N (B). Parasites were transfected with a CRISPR/Cas9 vector producing a single guide RNA (sgY328C, sgY483N or sgE545K) to target the Cas9 editing enzyme to 20-bp sites on wild-type *CPSF3* (see Fig 2). After cleavage by Cas9, homology-dependent repair from a 120-base donor oligonucleotide (Y328C, Y483N or E545K) resulted in incorporation of the specific SNP (shown in red). For clarity, only chromatograms of Y328 and Y483N are shown. E545K data is shown in Fig 2B.

 © 2017 The Authors

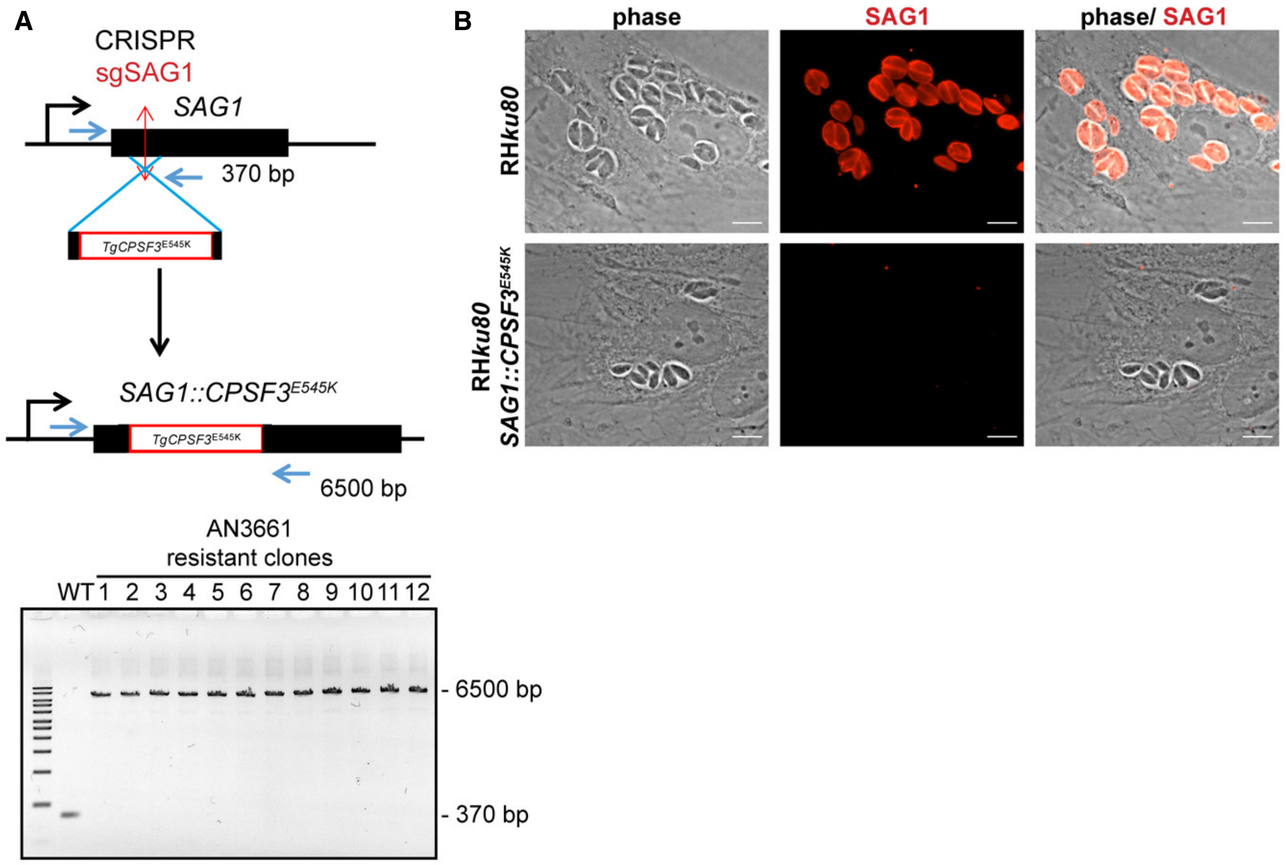

Figure EV3. Insertion of *CPSF3^{E545K}* (AN3661-resistant) in the *SAG1* locus.

A Schematic overview of the *SAG1* gene editing strategy with CRISPR/Cas9 and the *CPSF3^{E545K}* resistance cassette. Wild-type parasites (RH *ku80*) were transfected with the CRISPR/Cas9 vector, producing sgSAG1 RNA that targets Cas9 to the *SAG1* coding sequence. After cleavage by Cas9, homology-dependent repair was directed by a donor PCR amplicon encompassing *CPSF3^{E545K}* coding sequence with 5′ and 3′ regulatory sequences flanked by 60-bp sequences homologous to *SAG1*. Transfected parasites were selected in the presence of 5 μM AN3661. Insertion of the *CPSF3^{E545K}* cassette within *SAG1* was verified by PCR analysis in all clones using the indicated primers (in blue); bands at 6,500 bp indicate correct insertion of *CPSF3^{E545K}*, and the band at 370 bp is specific for wild-type *SAG1*.

B Detection of *SAG1* by immunofluorescence. The parental wild-type strain (RH *ku80*) and a *SAG1* mutant strain [RH *ku80 SAG1::CPSF3^{E545K}*, clone #1 in (A)] were stained using anti-SAG1 antibodies. Scale bars represent 10 μm.

© 2017 The Authors

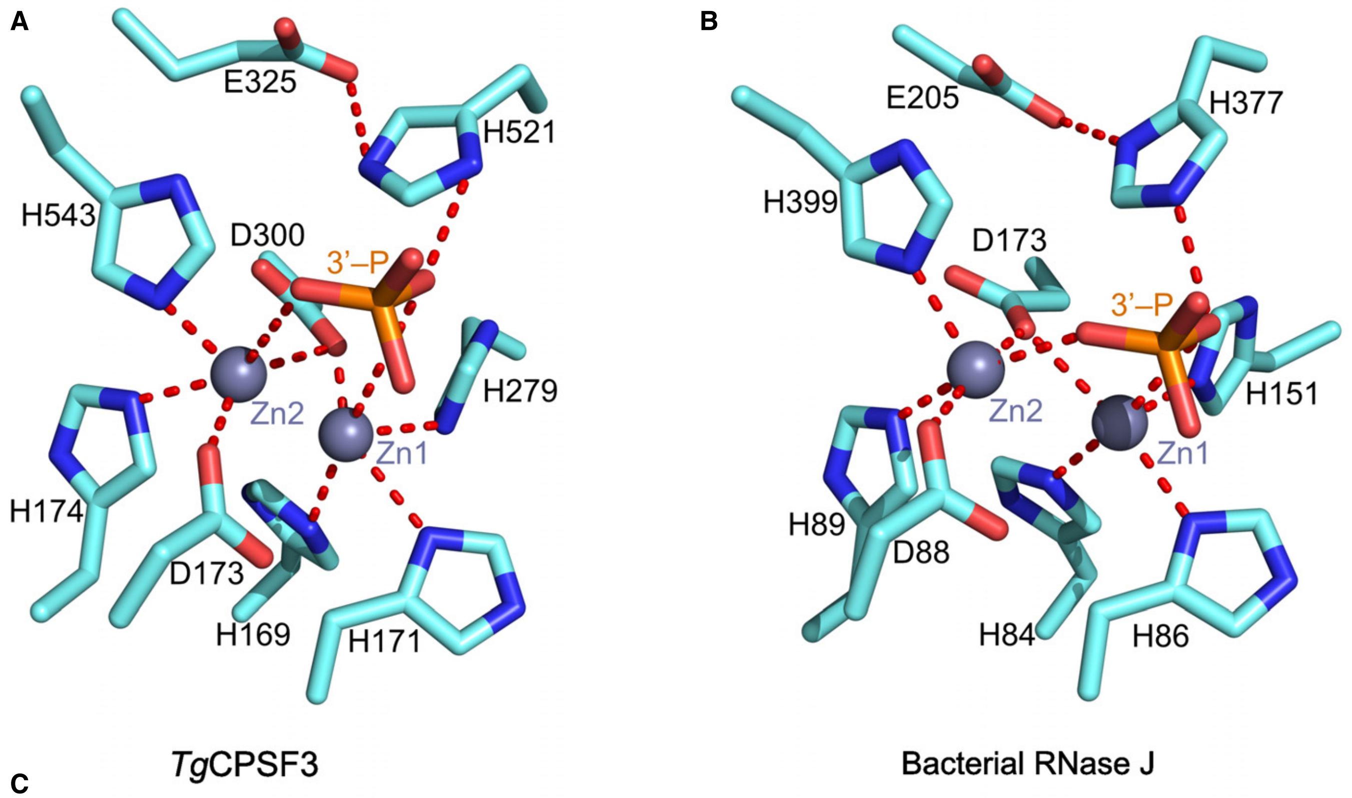

C

```
TgCPSF3  164VCLVTHFHLDHCGALP179...276GAGHVL-GACMFLIEIGGVRMLYTGDFS303
PfCPSF3  58.LCLITHFHMDHSGALP.73...200RAGHVI-GACMFLVEINNIRFLYTGDYS226
HsCPSF3  66.LLLISHFHLDHCGALP.81...155HAGHVL-GAAMFMIEIAGVKLLYTGDFS181
ScRNase  79.GIVLTHGHEDHIGGVP.94...148AVNHSIPDALAVAIRTPAGMVVHTGDFK175

TgCPSF3  321LLICESTYG329.......517SFSAHSDYQQTQEFIGKLKVPNVVLVHGERG525
PfCPSF3  245VLICEGTYG253.......444SFSAHSDFNQTKTFIEKLKCPNVVLVHGDKN472
HsCPSF3  200ILIIESTYG208.......392SFSAHTDYQQTSEFIRALKPPHVILVHGEQN422
ScRNase  201LLLADSTNA209.......373HVSGHASAGELLYFYNICRPKNLMPVHGEWR403
```

Figure EV4. Conservation of catalytic residues in *Toxoplasma gondii* CPSF3.

A, B Coordination of Zn atoms at the catalytic sites of the *T. gondii* CPSF3 model (A), same model as in Fig 3A, and *Streptomyces coelicolor* RNase J (B), PDB: 5A0T. The position of the phosphate of the 3′-mRNA at the cleavage position is shown for reference. Key interactions are shown as red dashed lines.

C Sequence alignment of regions constituting the catalytic sites of CPSF3 homologous proteins of *Toxoplasma gondii* (*Tg*), *Plasmodium falciparum* (*Pf*), *Homo sapiens* (*Hs*) and of RNase J from *Streptomyces coelicolor* (*Sc*).

 © 2017 The Authors
